# *Picrorhiza kurroa* Enhances *β*-Cell Mass Proliferation and Insulin Secretion in Streptozotocin Evoked *β*-Cell Damage in Rats

**DOI:** 10.3389/fphar.2017.00537

**Published:** 2017-08-22

**Authors:** Shiv Kumar, Vikram Patial, Sourabh Soni, Supriya Sharma, Kunal Pratap, Dinesh Kumar, Yogendra Padwad

**Affiliations:** ^1^Pharmacology and Toxicology Lab, Food and Nutraceuticals Division, CSIR-Institute of Himalayan Bioresource Technology Palampur, India; ^2^Academy of Scientific and Innovative Research New Delhi, India; ^3^Natural Product Chemistry and Process Development Division, CSIR-Institute of Himalayan Bioresource Technology Palampur, India

**Keywords:** streptozotocin, *β*-cell regeneration, insulin expression, glucose uptake, *P. kurroa*

## Abstract

Autoimmune destruction of insulin producing pancreatic β-cells leads to insulin insufficiency and hyperglycemia in type 1 diabetes mellitus. Regeneration of β-cells is one of the proposed treatment for type 1 diabetes and insulin insufficiency. *Picrorhiza kurroa* is a medicinal herb and is traditionally being used for the treatment of various diseases. Previous studies reported the hypoglycemic potential of *P. kurroa*. However, its potential role in β-cell induction in insulin secretion have not been fully investigated. Here, we characterized the hydro alcoholic extract of *P. kurroa* rhizome (PKRE) and further studied its β-cell regeneration and induction of insulin secretion potential in streptozotocin (STZ) induced diabetic rats as well as in insulin producing Rin5f cells. ^1^H-NMR revealed the presence of more than thirty metabolites including picroside I and II in PKRE. Further, we found that PKRE treatment (100 and 200 mg/kg dose for 30 days) significantly (*p* ≤ 0.05) protected the pancreatic β-cells against streptozotocin (STZ) evoked damage and inhibited the glucagon receptor expression (Gcgr) in hepatic and renal tissues. It significantly (*p* ≤ 0.05) enhanced the insulin expression and aids in proliferation of insulin producing Rin5f cells with elevated insulin secretion. Furthermore it significantly (*p* ≤ 0.05) increased insulin mediated glucose uptake in 3T3L1 and L6 cells. On the contrary, in diabetic rats, PKRE significantly (*p* ≤ 0.05) decreased high blood glucose and restored the normal levels of serum biochemicals. Altogether, our results showed that PKRE displayed β-cell regeneration with enhanced insulin production and antihyperglycemic effects. PKRE also improves hepatic and renal functions against oxidative damage.

## Introduction

Destruction of insulin producing *β*-cells is one of the serious chronic autoimmune disorders, characterized by infiltrating autoreactive lymphocytes, macrophages and other immune cells leading to diminished insulin secretion and hyperglycemia (Soltani et al., 2011). It is one of the most prevalent pediatric endocrine illnesses in developing nations and is termed as type 1 diabetes mellitus (T1DM). T1DM incidences increase globally by 2–5% per year (Poudel et al., 2015) and in India approximately 0.97 million children (below the age of 15) are affected (Kumar, 2015). T1DM generally manifests during early childhood and adolescence, potentially triggered by autoimmune, genetic, as well as environmental factors (Kakleas et al., 2015). It has been reported that 85–90% of *β*-cells are lost during onset of clinical symptoms, attributed to autoimmunity (Klinke, 2008). A meta-analysis study revealed that, 78% of insulitis occurs in children below 14 years, whereas 29% insulitis was diagnosed between 15 to 40 years of age. However, the severity of *β*-cells death decreased with age (Battaglia et al., 2015). Currently available antidiabetic drugs potentially sustain the blood glucose homeostasis with several adverse complications (Meier et al., 2015). Hence, alternative medications derived from medicinal herbs with potent hypoglycemic activity and minimal undesired complications are gaining importance. Ethnobotanical knowledge revealed that extracts and isolated molecules derived from medicinal plants have been widely used to treat type I and type II diabetes (Sheikh et al., 2015; Coelho et al., 2016; Devi et al., 2016).

Traditionally, roots and rhizomes of the herb *P. kurroa* found in Indian Himalayan region were widely used to treat fever, hepatitis, respiratory tract diseases, allergies and other inflammatory conditions (Dwivedi et al., 1992; Luper, 1998). Owing to the various pharmacological attributes of this medicinally important endangered herb, researchers developed efficient protocols for its *in vitro* mass multiplication and alternative agronomic practices (Nautiyal et al., 2001; Mahajan et al., 2016; Tanuja and Preeti, 2016). It is well reported that the pharmacological efficacy of this herb is due to kutkin, an active principal which comprises of iridoid glycosides (Picroside I and II) and kutkoside mainly localized in the roots and rhizome (Shitiz et al., 2013). Recently it was reported that iridoid glycosides potentially inhibit the formation of antiglycation products, resists hyperglycemia and regulates immunity *in vitro* as well as *in vivo* (Chen et al., 2016). Studies have shown that picroliv, a derivative of *P. kurroa* ameliorates dextran-sulfate-sodium-induced colitis in mice by attenuating the activity of SOD and myeloperoxidase. Further, it also reduces the transcript levels of TNF-α and IL-1*β* by inhibiting NF-κB expression (Zhang et al., 2012). *P. kurroa* rhizome extract exhibited promising antioxidant activity against indomethacin induced gastric ulcers in rats. Treatment significantly improved the SOD and catalase activity along with reduction in lipid peroxides (Ray et al., 2002). Preliminary observations by Joy and Kuttan (1999) revealed hypoglycemic effect of *P. kurroa* in alloxan induced diabetic rats, however, a systematic and detailed investigation on the potential of *P. kurroa* in curtailing insulin insufficiency is still lacking. Therefore present study was aimed to characterize the PKRE using metabolomic approaches and its *β*-cell proliferation efficacy, insulin gene expression as well as its secretion *in vitro* and *in vivo*. Study also investigates PKRE’s impact on oxidative damages in diabetic rats along with glucose uptake *in vitro.* Phytomolecular profiling results revealed the presence of more than thirty one major metabolites including picroside I and II in PKRE. Treatment effectively elevates the pancreatic *β*-cell mass *in vivo* with enhanced proliferation and insulin gene expression in Rin5f cells. Also it further enhances the glucose uptake in differentiated adipocytes and myotubes. Study also reports that PKRE improves the hepatic and renal complications associated with hyperglycemia and restores the normal functioning.

## Materials and Methods

### Chemicals and Reagents

Dexamethasone, insulin, protease inhibitor cocktail, rosiglita zone and 2-deoxy-2-[(7-nitro-2,1,3-benzoxadiazol-4-yl)amino]–glucose (2-NBDG), TBA, SRB and trysin were purchased from Sigma Aldrich, United States. Phenazine methosulphate (PMS), nitroblue tetrazolium (NBT), and nicotinamide adenine dinucleotide (NADH) were procured from Himedia labs, India. Hydrogen peroxide was purchased from MP biomedicals, United States. Kits for ALT, AST and ALP, albumin, BUN, CK analysis were purchased from ERBA (Germany). Anti-mouse insulin monoclonal antibody (Thermo Fischer Scientific, United States) and anti-rabbit glucagon receptor N-term polyclonal antibody (Abcam, United Kingdom) were used for the study. Analytical grade chemicals and solvents were used in the current study.

### Plant Material

Plant material of *P. kurroa* was collected from Holi-Nala village, district Chamba (Altitude 2800-3200 m) located in the Dhauladhar range of Himachal Pradesh, India. The plant material was authenticated and deposited in CSIR-IHBT, Palampur herbarium (PLP 11694).

### Extraction and Analysis of Samples

One kilogram *P. kurroa* (rhizomes) was extracted with 80% hydro alcohol and dried using rota evaporator at 45 ± 5°C to yield 21%. PKRE at 30 mg/mL concentration and standards of Mannitol, Picroside I and II at 5 mg/mL were prepared with NMR grade D_2_O and methanol-d_4_ (80:20) for NMR spectroscopic analysis. PKRE at 10 mg/mL and picroside I and II at 1mg/mL concentrations were prepared with HPLC grade methanol for LC-MS analysis respectively. The same extract was used for *in vitro* and *in vivo* studies.

### NMR Spectroscopy

PKRE was dissolved in 1 mL of D_2_O and methanol-d_4_ (80:20), 600 μL was transferred to 5 mm NMR tube. NMR spectra was recorded at 25°C on a 600 MHz Bruker AVANCE III-600 spectrometer (Bruker, Germany) operating at a proton NMR frequency of 600.30 MHz, while carbon at 150.94 MHz. Each ^1^H-NMR spectrum comprised of 64 scans, in 6 min and 50 s acquisition time with the following parameters: 0.16 Hz/point and relaxation delay (RD) = 5 s. A pre-saturation sequence was used to suppress the residual H_2_O/D_2_O/ methanol-d_4_ signal with low power selective irradiation at the H_2_O/methanol frequency during the recycle delay. FIDs were fourier transformed with LB = 0 Hz. The resulting spectra were manually phased and baseline was corrected and calibrated at 0.0 ppm, using Topspin (version 3.2, Bruker). Homonuclear (COSY) and heteronuclear spectroscopy (HMBC) based spectra were recorded on a 600.30 MHz (^1^H) and 150.94 MHz (^13^C) Bruker AVANCE III- spectrometer. The COSY spectra were recorded with 1.0 s relaxation delay, NS = 16, 8012.82 Hz spectral width in both dimensions while the window function was sine-bell (SSB = 0). Whereas, HMQC spectra were recorded with 1 s relaxation delay, NS = 64, 6009.61 Hz spectral width in F2 and 36,056.78 Hz in F1while window function of the HMQC was Q sine (SSB = 2.0). The HMBC spectra were recorded with the 1 s relaxation delay, NS = 64, 9615.38 Hz spectral width in F2 and 36230.55 Hz in F1. Same parameters as the HMQC spectra except for 36,056.78 Hz of spectral width in F2 were applied. The detailed study was done as described earlier (Kumar et al., 2016).

### HPTLC Fingerprint of PKRE

The HPTLC fingerprint study was carried out on both normal and reverse phase HPTLC plates according to Kumar et al. (2016) method. PKRE was dissolved in HPLC grade methanol at 10 mg/mL concentration. Four and eight microliters sample was used for HPTLC fingerprint on normal and reverse phase plates (Merck, Switzerland). The solvent system used as mobile phase was ethyl acetate:methanol:acetic acid (16:2:0.2; v:v:v) for normal phase and methanol:water:formic acid (64:36:0.4; v:v:v) for reverse phase (RP-18). The separated bands on the HPTLC plates were viewed using CAMAG Reprostar 3 and recorded at 366 nm after derivatization of NP-HPTLC plate with anisaldehyde sulpuric acid reagent followed by heating at 118°C for 5 min whereas reverse phase HPTLC (RP-18) was viewed and recorded at 254 nm for good separation of picrosides. CAMAG winCats^TM^ 1.4.1 was used for analysis.

### UPLC Based Quantification of Picrosides (Picroside I and II) in PKRE

PKRE was dissolved in UPLC grade methanol to yield a 10 mg/mL. The standard stock solutions (Picroside I and II; 1.0 mg/mL) were diluted to a series of suitable concentrations. An aliquot (0.5 μL) of the diluted solutions was injected into a Waters ACQUITY UPLC system (Waters, United Kingdom) with a Waters BEH C18 column, 1.7 μm (2.1 mm × 100 mm) coupled to a 1998 photodiode array (PDA) detector and used to scan at wavelength range of 200–490 nm for preparing seven-point calibration curves by plotting the peak areas versus amount (μg) of each analyte. The mobile phase consists of 0.1% HCOOH in H_2_O (solvent A) and C2H3N (solvent B) at constant flow-rate of 0.3 mL/min. The linear gradient profile with the following combinations (v/v) of solvent B was used [*t*(min), % B]: (0, 5), (0.5, 5), (9, 30), (12, 30), (13, 5), (15, 5) over the 15 min time period. The peaks were recognized by comparing with the reference standards retention time and UV spectra. The 270 nm wavelength was selected for the quantification (Kumar et al., 2016).

### *In Vivo* Experiments

#### Animals

Young, male Wistar rats (*n* = 30), 6–8 weeks old, weighing 100–120 *g* were obtained from experimental animal facility of CSIR-IHBT, Palampur. Animals were acclimatized at standard laboratory conditions (12 h light/dark cycle; 50–60% humidity and 22 ± 2°C temperature) prior to the experiment. Animals were caged in polypropylene cages (*n* = 6) and fed with pelleted chow and water *ad libitum* throughout the experiment. The study was approved by Institutional Animal Ethics Committee, CSIR-IHBT, Palampur (Approval number; IAEC/IHBTP3-2013).

#### Oral Glucose Tolerance Test

In order to evaluate the effect of PKRE on peripheral blood glucose levels, OGTT was performed. Overnight fasted rats were randomly categorized into four groups. Group I; administered with distilled water, Group II; administered with GLB (10 mg/kg body weight dose; Husain et al., 2009; Rehman et al., 2014), while Group III and IV were administered with PKRE at 200 and 400 mg/kg dose, respectively. After 30 min of PKRE or GLB treatment, animals were administered with glucose (2 g/kg dose) orally. Blood glucose levels were measured at varying time points using glucometer (Contour TS, Blood glucose monitoring kit, Bayer Healthcare).

#### Induction of T1DM and Experimental Design

Rats maintained at fasting condition overnight were injected with a single dose of freshly prepared STZ (Calbiochem, United States; 50 mg/kg dose) in 0.1 M citrate buffer (pH 4.5) intraperitoneally while control animals were injected with citrate buffer alone (Rao et al., 2013; Tripathi and Kohli, 2014; Altinoz et al., 2015). After 1 week, fasting blood glucose was measured using glucometer and animals with hyperglycemic condition at blood glucose levels above 300 mg/dL were taken further for the study. Animals were categorized into five different groups (*n* = 6) as per experimental regime. Group I; normal control rats, group II; STZ intoxicated rats, group III; diabetic rats administered with standard GLB at the dose of 5 mg/kg daily for 30 days (Cheng et al., 2013; Tripathi et al., 2015; Panjeshahin et al., 2016), group IV; diabetic rats administered with 200 mg/kg dose of PKRE for 30 days and group V; diabetic rats administered with 400 mg/kg dose of PKRE for 30 days. The PKRE doses were finalized on the basis of previous studies by Joy and Kuttan (1999), Husain et al. (2009), Husain et al. (2014). Doses were administered orally using intragastric tube by trained expert in experimental animal facility. Parameters such as body weight and blood glucose were measured at 0, 15th and 30th day of the study. At the end of experiment, animals were euthanized by CO_2_ asphyxiation and dissected to collect blood and vital organs for further investigations.

#### Serum Biochemical Estimations

Blood from each animal was collected prior to dissection via retro-orbital plexus puncture and allowed to clot at room temperature followed by centrifugation at 5000 rpm for 15 min. Serum thus collected was used for biochemical estimations. The levels of ALT, AST, ALP, albumin, CK and BUN were quantitated using commercially available Erba diagnostic kits as per manufacturer’s protocols in Erba XL 200 analyzer (Erba Mannheim, Germany).

#### Superoxide Dismutase and Catalase Measurement

Hepatic and renal tissues were homogenized in 0.1 M ice cold phosphate buffer saline (PBS; pH 7.3) using homogenizer (IKA T-10 basic, Germany), and resultant crude tissue homogenate was centrifuged at 10,000 rpm and 4°C for 20 min. The supernatants obtained were subjected to the estimation of SOD and CAT activities. Results were expressed as units per mg tissue for SOD and CAT as described by Sharma et al. (2016).

#### Malondialdehyde Quantification

Malondialdehyde levels were measured in hepatic and renal tissues using TBA reaction method as described by Patial et al. (2015). Homogenates were prepared from 200 mg tissue in ice cold 0.1M PBS (pH 7.4) and resultant crude homogenates were centrifuged at 10,000 rpm and 4°C for 10 min. The supernatants obtained were used for MDA estimation. For the assay, 1 mL supernatant was maintained at 37°C for 3 h. Reaction was halted by adding 1 mL of 10% TCA (w/v). The contents were allowed to precipitate at room temperature followed by centrifugation at 2000 rpm for 10 min. 1 mL of supernatant was mixed with same volume of 0.67% TBA (w/v), boiled in water bath for 10 min, allowed to cool down and the absorbance was measured at 535 nm, using microplate reader (Synergy; BioTek, United States).

#### Histopathology

Portions of pancreas were fixed in fixative (10% neutral buffered formalin) for histopathological evaluation. Briefly, tissue sections were processed for dehydration, clearing and were embedded in paraffin. Five micrometer sections were obtained using microtome (Finesse me; Thermo Fischer Scientific, United States) and were further deparaffinized, dehydrated and stained with hematoxylin (Himedia labs). Sections were counterstained with eosin, mounted and were analyzed for detection of STZ evoked cellular damage using bright field microscope (Olympus BX53F microscope, Japan).

#### TUNEL Assay

For the detection of apoptosis in pancreas, 5 μm paraffin embedded sections were used. Endogenous peroxidases were blocked by treating sections with H_2_O_2_ (0.3%, v/v) in PBS before enzymatic labeling. Fragmented DNA was labeled with horseradish peroxidase (HRP) conjugated biotinylated nucleotide at 3’OH end using the terminal deoxynucleotidyl transferase recombinant enzyme by following manufacturer’s protocol (Promega, United States). Detection was performed using H_2_O_2_ and diaminobenzidine (DAB) substrate. Further sections were counterstained with Mayer’s hematoxylin, dehydrated, cleared and mounted. Observations for cellular apoptosis were made under Olympus BX53F bright field microscope (Japan).

#### Immunohistochemistry

The 5 μm paraffin embedded pancreas sections were mounted on poly-L-lysine overlayed slides, deparaffinized with xylene and hydrated. Antigen retrieval was achieved by treating sections with sodium citrate buffer. Quenching of endogenous peroxidases was accomplished using BLOXALL blocking solution (ImmPRESS excel staining kit, United States) and exposed sites were blocked by treating sections with horse serum (2.5%) for 20 min in a humidified chamber. Blocked sections were probed overnight with anti-mouse insulin monoclonal antibody (Thermo Fischer Scientific, United States) in dilution of 1:100 and anti-rabbit glucagon receptor N-term polyclonal antibody (Abcam, United Kingdom) in dilution of 1:200. Unbound primary antibodies were removed by PBS wash followed by incubation with HRP-conjugated anti-mouse and anti-rabbit secondary antibodies. The sections were PBS rinsed twice and incubated with diaminobenzidine (DAB) substrate. Developed sections were counterstained with Mayer’s hematoxylin, dehydrated, and then mounted. Examinations were performed under Olympus BX53F bright field microscope for protein expression studies.

### Cell Culture

The rat insulinoma (Rin5f), rat myoblast (L6) and mouse preadipocyte (3T3L1) cell lines were procured from ATCC, United States. Rin5f cells were grown in RPMI-1640, whereas L6 and 3T3L1 cells were grown in Dulbecco’s Modified Eagle’s medium (DMEM). The culture medium was supplemented with 10% (v/v) heat inactivated FBS (Life Technologies, United States) and 1% (v/v) antibiotic-antimycotic (Life Technologies, United States). Cells were passaged once a week after trypsinization (0.25% trypsin containing 0.2% EDTA) and residual medium was replaced with fresh medium twice a week. Cultures were maintained in CO_2_ incubator (Thermo Fischer scientific, United States) with 95% humidity, 5% CO_2_ at 37°C. After second passage cells were used for experiments.

### Cell Viability Assay

Effect of PKRE on the viability of Rin5f, L6 and 3T3L1 cells was determined by SRB assay (Rana et al., 2016). Briefly, confluent monolayer of cells was trypsinized, counted and seeded in a flat bottom 96 well plate at density of 2 × 10^4^ cells/well with culture medium. After 24 h, residual medium was replaced and cells were incubated with new culture medium containing indicated concentrations (viz, 6.25, 12.5, 25, 50, 100, 200, 300, 400, and 500 μg/mL) of PKRE for 48 h. After desired incubation, cells were fixed with 50% (w/v) cold TCA and then plates were kept at 4°C for 1 h. Following 1 h incubation, plates were washed with distilled water four times and allowed to dry at room temperature. Dried plates were stained with 0.4% (w/v) SRB in 1% (v/v) acetic acid for 30 min in dark. Excess and unbound SRB dye was removed by repeated washing with 1% (v/v) acetic acid and plates were allowed to air dry. Absorbance was measured at 540 nm using microplate reader by solubilizing the bound dye with 10 mM (w/v) Tris HCl.

### *In Vitro* Insulin Secretion Assay

Rin5f cells were seeded in 6 well plates at density of 2 × 10^5^ cells/well and allowed to grow for 4–5 days to attained 80–90% confluency. Cells were washed twice with Kreb’s-Ringer bicarbonate buffer (KRB: 115 mM, NaCl; 4.7 mM, KCl; 1.28 mM, CaCl_2_,1.2 mM, KH_2_PO_4_; 1.2 mM, MgSO_4_; 24 mM, NaHCO_3_; 10 mM Hepes-free acid, 1 g/L bovine serum albumin, 1.1 mM glucose; pH 7.4) and then pre-incubated in the same buffer for 60 min at 37°C. After incubation, cells were again incubated with fresh KRB buffer containing 25 mM glucose with or without PKRE for 90 min. Post incubation supernatants were collected and insulin secretion was measured using enzyme linked immunosorbent assay (ELISA) performed as per manufacturer’s protocol (Raybiotech, United States).

#### Total RNA Isolation and qRT-PCR

Rin5f cells were seeded in 60 mm cell culture petri dish and allowed to grow upto 80–90% confluency. Cells were incubated in KRB buffer containing 1.1 mM glucose for 60 min followed by incubation in KRB buffer containing 25 mM glucose with or without PKRE for 90 min. Cells were washed twice with PBS and total RNA was extracted using Qiagen RNeasy mini kit (Qiagen, Germany) following manufacturer’s protocol. RNA was quantified spectrophotometrically using nanodrop (Thermo Fisher Scientific, United States) and purity of RNA was assessed as 260/280 nm absorbance ratio. The integrity of RNA was confirmed by identifying 18 and 28 s RNA bands on 1% agarose gel. One step qRT-PCR was performed using QuantiFast^®^ SYBR^®^ Green RT-PCR kit (Qiagen, Germany) according to manufacturer’s instructions. Primers were designed using the Primer Express software (Applied Biosystems). For insulin; forward primer, 5′-GCCCAGGCTTTTGTCAAACA-3′ and reverse primer, 5′-AAACCACGTTCCCCACACA-3′ and for GAPDH; forward primer, 5′-GGTGGACCTCATGGCCTACA-3′ and reverse primer, 5′-CAGCAACTGAGGGCCTCTCT-3′ were used. RNA template at concentration of 50 ng was added to the final reaction volume of 10 μL. The PCR reactions were carried out in 96 well plates using the Step one Plus^TM^ Real Time PCR system (Applied Biosystems, United States). GAPDH was used as housekeeping gene to normalize the expression of target gene.

### Fluorescence analysis of 2-[N-(7-Nitrobenz-2-oxa-1,3-diaxol-4-yl)amino]-2-Deoxyglucose (2-NBDG) Uptake by Flow Cytometry in 3T3L1 and L6 Cells

L6 cells were seeded in 6 well plate at 1 × 10^5^ cells/well and allowed to differentiate into myotubes for 5 days in DMEM supplemented with 2% horse serum (Himedia labs, India). The medium was replaced on alternate days. On the 5th day of seeding the residual medium was replaced with fresh medium with or without PKRE concentrations (6.25, 12.5, 25, 50, and 100 μg/mL) for 24 h. Rosiglitazone (10 μM) was used as positive control. After 24 h, cells were washed twice with KRB buffer (Sigma Aldrich, United States) and then pre-incubated in the same buffer for 30 min. Starved cells were treated with 100 μM 2-NBDG (Fluorescent analog of glucose) for 1 h. After incubation, cells were washed twice with ice cold KRB buffer, trypsinized, resuspended in same buffer and subjected to flow cytometric analysis using AMNIS ImageStream^®X^ MK II Imaging flow cytometer (Merck, United States). Data acquired as 5000 single circular events.

3T3L1 cells were plated at a density of 1 × 10^6^ cells/well in 6 well plate and allow them to grow. At 2 days post confluence stage, cells were induced to differentiate in differentiation medium supplemented with 0.5 mM 3-isobutyl-1-methylxanthine (IBMX), 5 μg/mL insulin, 250 nM Dexamethasone in DMEM and 10% FBS for 48 h. After 2 days, the residual medium was replaced with maintenance medium containing DMEM with 10% FBS and 5 μg/mL insulin. This medium was replaced alternatively upto 9 days. At the end of 9th day, cells were treated with different concentrations of PKRE (6.25, 12.5, 25, 50, and 100 μg/mL) and rosiglitazone at 10 μM concentration for 24 h. On next day, treatment medium was removed and cells were incubated in serum free low glucose medium with or without PKRE for 3 h and further stimulated by adding insulin (5 μg/mL) for 10 min. Stimulated cells were treated with 100 μM 2-NBDG for 1 h, 2-NBDG labeled cells were trypsinized, centrifuged at 125 g for 7 min and the supernatant were decanted whereas cell pellet were suspended in ice cold KRB buffer for data acquisition. Flow cytometry was performed using AMNIS ImageStream^®X^ MK II Imaging flow cytometer (Merck, United States) and data were analyzed using INSPIRE ImageStream system software. Data acquired as 5000 single circular events.

### Statistical Analysis

Results are expressed as mean ± standard error mean (SEM). Differences were analyzed using GraphPad software by one way ANOVA followed by Dunnett’s multiple comparison range test. Values with *p* ≤ 0.05 were considered as statistically significant.

## Results

### Phytochemical Profiling of PKRE

**FIGURE 1:**
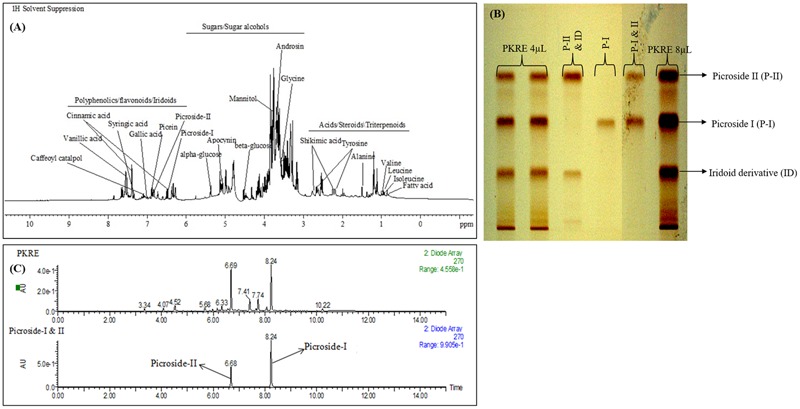
**(A)** Chemical profiling of PKRE, **(B)** RP-HPTLC fingerprint of PKRE **(C)** UPLC-DAD chromatogram of PKRE and standard mixture (Picroside I; 8.24 and Picroside II; 6.68).

Thorough phytomolecular characterization of PKRE was carried out by NMR based metabolomic approach and further validated by HPTLC and UPLC. The obtained fingerprint deciphers the identification of major metabolites and revealed the structural and functional diversity of *P. kurroa*. ^1^H-NMR spectra of PKRE is divided into three major regions such as amino acids/organic acids/cucurbitacins, sugars/polyols and phenolics/iridoids/flavonoids (**Figure 1A**). Moreover, the intensity of signals remained very low and overlapped for few regions. Hence, they were difficult to interpret. Therefore, 2D NMR analysis techniques such as ^1^H–^1^H COSY (homonuclear correlation), HMQC, and HMBC were employed along with 1D NMR to overcome these problems. The NMR based chemical profiling of PKRE was shown in **Figure 1A** (Supplementary Figure S1a and Table S1). 1D and 2D NMR based chemical profiling highlighted the presence of 31 metabolites belonging to different classes. The HPTLC fingerprint confirmed the presence of picroside I and II in majority, (**Figure 1B**).

The UPLC-DAD based analysis also revealed the presence of picrosides. The PDA chromatogram of PKRE and standards was depicted in **Figure 1C** while the calibration curves of picroside I and II were used for the quantification (Supplementary Figures S1b,c). The total picrosides (picroside I and II) were found 13.87% while the individual picroside I and II were 9.57 and 4.30%, respectively.

### PKRE Ameliorate Oral Glucose Tolerance

**FIGURE 2:**
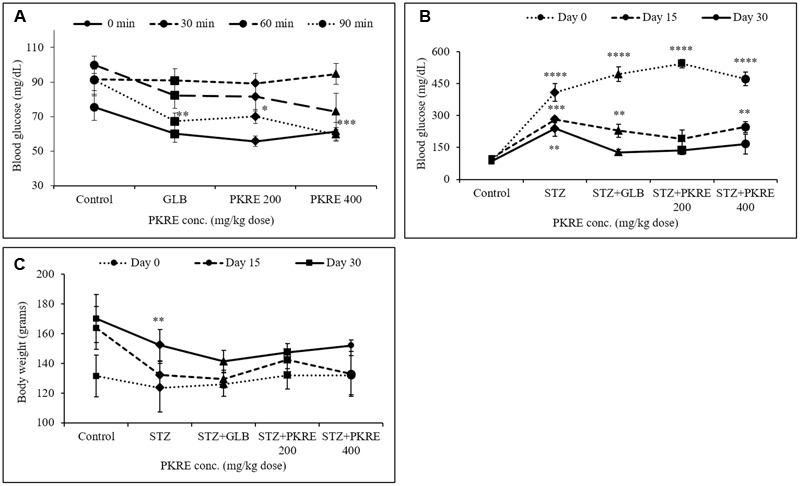
**(A)** PKRE treatment ameliorated glucose tolerance in OGTT effectively after 90 min of glucose load in normal Wistar rats. Blood glucose levels were measured at 0, 30, 60, and 90 min after oral glucose load **(B)** PKRE treatment effectively lowered high blood glucose level in STZ intoxicated rats at both 200 and 400 mg/kg doses respectively. Fasting blood glucose was measured at 0, 15, and 30 days of experiment using kit **(C)** Effect of PKRE treatment on the body weights of different experimental groups recorded at 0, 15 and 30 days. Data are expressed as mean ± SEM (*n* = 6). ^∗∗^*p* < 0.01, ^∗∗∗^*p* < 0.001, and ^∗∗∗∗^*p* < 0.0001 indicate significant difference in groups compared to normal control group.

Amelioration of glucose tolerance obtained from integrated glucose to insulin response that maintains the normal glycemic conditions (Anhê et al., 2015). OGTT was performed to check the hypoglycemic potential of bioactive molecules. Results suggest that, initial blood glucose was 61.4 mg/dL and 55.6 mg/dL in PKRE assigned groups in comparison to normal control (75.4 mg/dL) and GLB assigned group (60 mg/dL). After 30 min, blood glucose levels reached at peak with 89.2 mg/dL at 200 mg/kg and 94.6 mg/dL at 400 mg/kg in PKRE treated groups compared to normal control group (91.4 mg/dL) and GLB treated group (90.8 mg/dL). Further, improvement was observed in the glucose tolerance and after 90 min significant response was recorded. PKRE significantly lowered the blood glucose to 70 mg/dL (*p* < 0.05) at 200 mg/kg and 59.6 mg/dL (*p* < 0.001) at 400 mg/kg dose when compared with normal control group (91.2 mg/dL) after 90 min. However, GLB treatment also effectively lowered the blood glucose to 67.2 mg/dL, (*p* < 0.01) after 90 min of glucose loading (**Figure 2A**).

### PKRE Improves Hyperglycemia and Weight Loss toward Normalcy

Impaired insulin secretion results from destruction of pancreatic *β*-cells that disturbs glucose homeostasis (Cerf, 2013). Increased blood glucose leads to serious metabolic complications and oxidative stress leading to cellular damages (Asmat et al., 2016). PKRE treatment for 15 days, significantly lowered the blood glucose levels to 190.8 mg/dL and 246 mg/dL (*p* < 0.01) in 200 and 400 mg/kg dose group respectively when compared to STZ intoxicated group (281.8 mg/dL, *p* < 0.001). After 30 days, PKRE treatment significantly (*p* < 0.0001) lowered the blood glucose levels to 136 mg/dL and 166 mg/dL in 200 and 400 mg/kg dose treated group in contrast to STZ intoxicated group (239.4, *p* < 0.01). Similar results were observed for GLB treated group (127.2 mg/dL) after 30 days. Blood glucose levels of STZ intoxicated group remained higher (239.4 mg/dL) till the end of experiment (**Figure 2B**).

STZ, GLB, and PKRE treatment doesn’t exert much effect on the body weight of control and diabetic rats throughout the experiment. At 0 day, 132 g body weight was recorded for both PKRE treatment groups, whereas 131.4, 123.5, and 126 g were recorded for normal control, STZ intoxicated and GLB treatment groups respectively. Further, increase in the body weight of animals was observed after treatments. After 15 days, it was recorded to 142.4 g in 200 mg/kg and 132.8 g in 400 mg/kg PKRE treated groups as compared to control group (163.8 g). The increase was recorded to 132.2 g for STZ intoxicated group and 129.4 for GLB treated group after 15 days. Further it was found that after 30 days, 147.4 and 152 g weight was recorded for 200 and 400 mg/kg PKRE treated groups respectively. Whereas, 152.25 g (*p* < 0.01) was recorded for STZ intoxicated group and 141.2 g for GLB treated group in contrast to control group (170 g) (**Figure 2C**).

### PKRE Treatment Maintained the Normal Levels of ALT, AST and ALP

**FIGURE 3:**
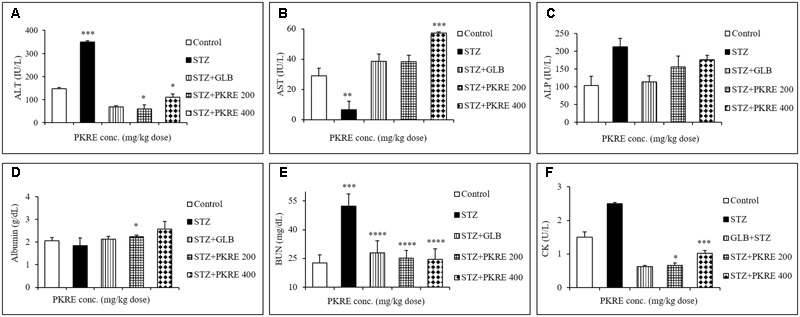
PKRE effectively improved the hepatic and renal function markers in streptozotocin intoxicated diabetic rats. ALT, AST and ALP **(A–C)** were measured for hepatic functioning whereas serum albumin, BUN and CK **(D–F)** were measured for renal functioning in serum samples of different experimental groups. Data are expressed as mean ± SEM (*n* = 6). ^∗^*p* < 0.05, ^∗∗^*p* < 0.01, ^∗∗∗^*p* < 0.001 and ^∗∗∗∗^*p* < 0.0001 indicate significant difference in groups compared to normal control group.

An elevated serum enzymes ALT, AST and ALP are the markers of hepatic damage and indicates the hepatotoxic effect of STZ (Srinivasan et al., 2014). ALT catalyzes the alanine cycle and its enhanced level suggests the existence of hepatic disorders. In the present study, increased level of ALT (350 IU/L, *p* < 0.001) was measured in STZ intoxicated group compared to its control group (147.3 IU/L). On the other hand PKRE treatment effectively lowered the ALT levels to 60.0 IU/L (*p* < 0.05) and 110.1 IU/L in 200 and 400 mg/kg dose groups respectively. However, GLB treatment also efficiently lowered the ALT level to (67.8 IU/L, *p* < 0.05) (**Figure 3A**). AST is one of the important enzyme in amino acid metabolism and also a marker of hepatic health. In STZ intoxicated group, the serum level of AST decreased significantly (6.6 IU/L, *p* < 0.01) compared to normal control group (28.9 IU/L), but PKRE administration restored the AST levels to 38.3 IU/L in 200 mg/kg and 57.1 IU/L (*p* < 0.001) in 400 mg/kg treated groups. Further, GLB treatment also increased the AST to 38.6 IU/L (**Figure 3B**). On the other hand, serum ALP levels tend to increase due to its increased cellular synthesis during biliary stress. Leakage of ALP in bloodstream increases in hepatic abnormalities (Ashraf et al., 2013). The levels of ALP increased to 212.7 IU/L in STZ intoxicated group in comparison to control group 103.0 IU/L, whereas PKRE treatment lowered the serum ALP levels to 155.8 IU/L in 200 mg/kg and 175.8 IU/L in 400 mg/kg dose treated groups. GLB also effectively reduced the ALP level to 113.8 IU/L (**Figure 3C**).

### PKRE Treatment Improves Serum Albumin, BUN and CK Levels

Serum albumin, BUN and CK levels are significantly altered in hyperglycemic situation as a result of renal damage, protein catabolism, extracellular dehydration and lack of glucose metabolism (Go et al., 2015). Serum albumin is the most abundant plasma protein imparting several physiological attributes including antioxidant property. Hyperglycemia induces the formation of advanced glycation end products (AGEs) that binds with albumin protein and significantly reduces its level (Raghav and Ahmad, 2014). The STZ intoxicated group showed reduced levels of serum albumin (1.8 g/dL) compared to normal control group (2.05 g/dL). PKRE treatment effectively enhanced the serum albumin level to 2.2 g/dL (*p* < 0.05), 2.5 g/dL at 200 and 400 mg/kg dose treated groups respectively whereas GLB also increased albumin level to 2.1 g/dL (**Figure 3D**). BUN and CK are the nitrogenous wastes and their increased serum levels are the markers of renal damage. Hyperglycemia mediated reactive oxygen species (ROS) affects glomerular filtration rate, which contributes in the accumulation of nitrogenous wastes in the blood stream (Altinoz et al., 2015). BUN was found increased in STZ intoxicated group (52.4 g/dL) as compared to control group (22.5 g/dL). PKRE treatment significantly (*p* < 0.0001) reduced the levels to 25 g/dL in 200 mg/kg and 24.4 g/dL in 400 mg/kg dose treated groups, GLB also brought down the BUN level to 27.9 g/dL (**Figure 3E**). CK levels were also found elevated in diabetic rats (2.5 U/L) compared to control group (1.5 U/L). PKRE significantly decreased the CK level to 0.66 U/L (*p* < 0.05) and 1.02 U/L (*p* < 0.001) in 200 and 400 mg/kg treated groups. GLB also potentially reduced the CK level to 0.62 U/L (**Figure 3F**).

### PKRE Improves Hepatic and Renal Functions by Normalizing Antioxidant Parameters

**FIGURE 4:**
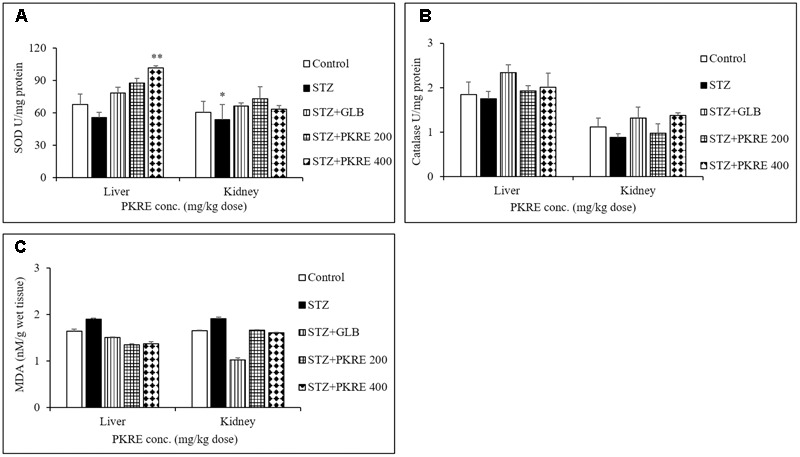
PKRE treatment ameliorated the activity of SOD **(A)** catalase **(B)** and simultaneously inhibited the MDA production **(C)** in hepatic and renal tissue homogenates of different experimental groups. Data are expressed as mean ± SEM (*n* = 6). ^∗^*p* < 0.05, ^∗∗^*p* < 0.01 indicate significant difference in group compared to normal control group.

Hyperglycemia induced oxidative stress results in reduced antioxidant levels and increased free radicals (Florence et al., 2014). These free radicals damages cellular components and triggers specific signaling pathways leading to secretion of inflammatory cytokines (Naowaboot et al., 2009). In the present study, levels of SOD, CAT and MDA were measured in the hepatic and renal tissues. SOD decreases the intracellular superoxide anion concentration by distmutating superoxide anion to hydrogen peroxide and oxygen (Ren et al., 2015). Reduced SOD levels were measured in hepatic (55.5 U) and renal tissues (53.5 U, *p* < 0.05) in STZ intoxicated group compared to control group at 67.7 U and 60.5 U respectively. Treatment with PKRE increased the SOD levels to 87.4 U and 73 U at 200 mg/kg dose, whereas significant (*p* < 0.01) increased in SOD levels (101.8 U and 63.2 U) were measured at 400 mg/kg dose treatment groups. GLB also restored the SOD levels to 78.2 U and 66.1 U in both tissues respectively (**Figure 4A**). CAT, an endogenous enzyme catalyzes the decomposition of hydrogen peroxide to less reactive oxygen and water, whereby reducing the cellular oxidative stress (Ren et al., 2015). STZ intoxicated group characterized by decreased CAT levels upto 1.7 U in hepatic and 0.8 U in renal tissues when compared with control group at 1.8 U and 1.1 U respectively. Administration of PKRE at 200 mg/kg dose increased CAT levels to 1.9 U and 0.9 U, which further elevated upto 2.0 U in hepatic and renal tissues at 400 mg/kg dose treated groups. Further, GLB treated group also showed the elevated levels of CAT to 2.3 U and 1.3 U respectively (**Figure 4B**). MDA, a highly reactive species and a marker of cellular oxidative stress results from lipid peroxidation of polyunsaturated fatty acids (PUFA). This affects membrane fluidity, integrity and cellular functions (Adefegha et al., 2014). Elevated level of MDA to 1.9 nM was measured in STZ intoxicated group in contrast to control group (1.6 nM) for both the tissues. Further, PKRE treatment effectively lowered the levels to 1.3 and 1.6 nM at both treatment doses respectively compared to STZ intoxicated group. GLB also potentially reduced the MDA level to 1.50 and 1.01 nM in hepatic and renal tissues respectively (**Figure 4C**).

### PKRE Protects Pancreatic *β*-Cells from STZ Evoked Cellular Damages and Enhances Insulin Expression

**FIGURE 5:**
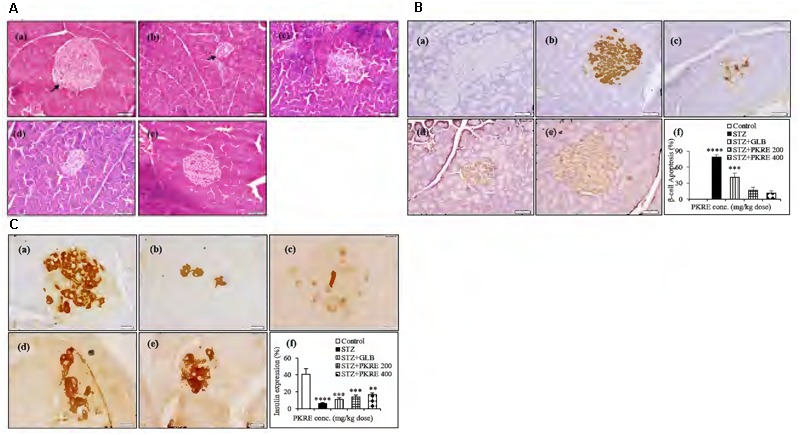
PKRE treatment significantly protects *β-*cells from STZ evoked cellular damage and enhanced the insulin production in diabetic rats. **(A)** Representative histopathological photographs showing STZ evoked *β*-cells alterations in different experimental groups detected by H and E staining. (a) Normal group, (b) Streptozotocin group, (c) GLB control, (d) PKRE 200 mg/kg and (e) PKRE 400 mg/kg. **(B)** Microscopic images of TUNNEL assay in various experimental groups for apoptosis assessment revealed the protective effect of PKRE in diabetic rats. (a) Normal group, (b) Streptozotocin group, (c) GLB control, (d) PKRE 200 mg/kg and (e) PKRE 400 mg/kg. **(C)** Representative Immunohistochemistry images of insulin expression in different experimental groups. PKRE treatment increased the insulin expression in diabetic rats. (a) Normal group, (b) Streptozotocin group, (c) GLB control, (d) PKRE 200 mg/kg and (e) PKRE 400 mg/kg. Data are expressed as mean ± SEM (*n* = 6). ^∗∗^*p* < 0.01), ^∗∗∗^*p* < 0.001 and ^∗∗∗∗^*p* < 0.0001 indicate significant difference in groups compared to normal control group.

Insulin deficiency in STZ induced hyperglycemia is a consequence of pancreatic *β*-cells damage by infiltrating autoreactive lymphocytes (Zhao et al., 2015). This results into elevated blood glucose which further leads to metabolic alterations. It is reported that regeneration of *β*-cell mass reverses the hyperglycemic effects in STZ intoxicated diabetic rats (Soltani et al., 2011). STZ evoked destruction of pancreatic *β*-cells with increased oxidative stress mimics the pathophysiology of T1DM. Histopathological analysis revealed that islets of STZ intoxicated rats showed cellular damage, cytoplasmic vacoulations, eosinophilia and pyknotic nuclei compared to normal control group that exhibited normal pancreatic architecture. PKRE exhibits marked protective effect against STZ evoked *β*-cell damage and islet degeneration at both the treatment doses (**Figure 5A**). Further, apoptosis in the pancreatic *β*-cells was detected through *in situ* TUNEL assay. STZ evoked the *β*-cells death and further reduction in β-cell mass is characterized by the detection of apoptotic bodies (79%, *p* < 0.0001). The apoptotic bodies were less in GLB treated group (41%, *p* < 0.001). However, the extent of apoptosis was very less in PKRE treated groups and found upto 17 and 11% at both doses respectively compared to normal control group with no such detections (**Figure 5B**). Furthermore, the expression of insulin in pancreatic *β*-cells was evaluated through IHC. The normal control group was found to have highest expression of insulin upto 40%, however, it was found drastically less in STZ intoxicated group (6.1%, *p* < 0.0001). In contrast, GLB (10.6%, *p* < 0.001) and PKRE treated groups showed the enhanced insulin expression in *β*-cells as compared to STZ intoxicated group. PKRE significantly enhances the insulin expression upto 13.9% (*p* < 0.001) and 16.4% (*p* < 0.01) at 200 and 400 mg/kg doses respectively (**Figure 5C**).

### PKRE Treatment Inhibits the Expression of Hepatic and Renal Gcgr

**FIGURE 6:**
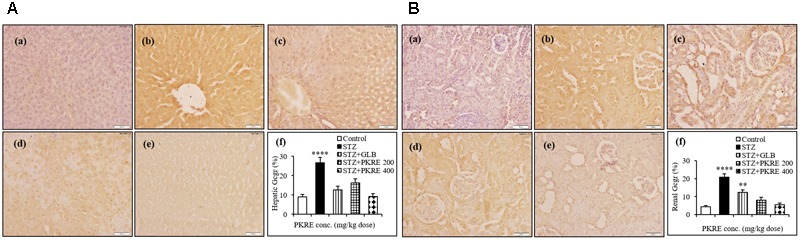
Inhibitory effect of PKRE on Glucagon receptor (Gcgr) expression in hepatic **(A)** and renal **(B)** tissues of different experimental groups. Representative immunohistochemistry images showed the Gcgr expression in various animal groups. (a) Normal group, (b) Streptozotocin group, (c) GLB control, (d) PKRE 200 mg/kg and (e) PKRE 400 mg/kg. Data are expressed as mean ± SEM (*n* = 6). ^∗∗^*p* < 0.01 and ^∗∗∗∗^*p* < 0.0001 indicate significant difference in groups compared to normal control group.

Evidence demonstrates that inhibition of *in vivo* glucagon signaling leads to hypoglycemia. Glucagon binds to its receptors, resulting in glucose release with enhanced gluconeogenesis and glycogenolysis. Gcgr are widely distributed in hepatic, renal and other tissues (Jiang and Zhang, 2003). IHC results revealed that hepatic Gcgr expression increases significantly (*p* < 0.001) to 26.5% in STZ intoxicated group when compared with normal control (9.05%). PKRE on the other hand effectively brought down the Gcgr expression to 16.3 and 9.3% at 200 and 400 mg/kg doses, respectively. GLB treatment also brought down the Gcgr expression to 12.6% (**Figure 6A**). Similarly in renal tissues, significant level of expression upto 20.9% (*p* < 0.0001) was found in STZ intoxicated group as compared to control (4.3%). PKRE administration effectively inhibited the Gcgr expression upto 8.1 and 5.4% at 200 and 400 mg/kg dose treatment group. Further, GLB treatment also inhibited the Gcgr expression to 12.4% (*p* < 0.01) (**Figure 6B**).

### PKRE Proliferates Rin5f cells with Enhanced Insulin Gene Expression and Insulin Secretion

**FIGURE 7:**
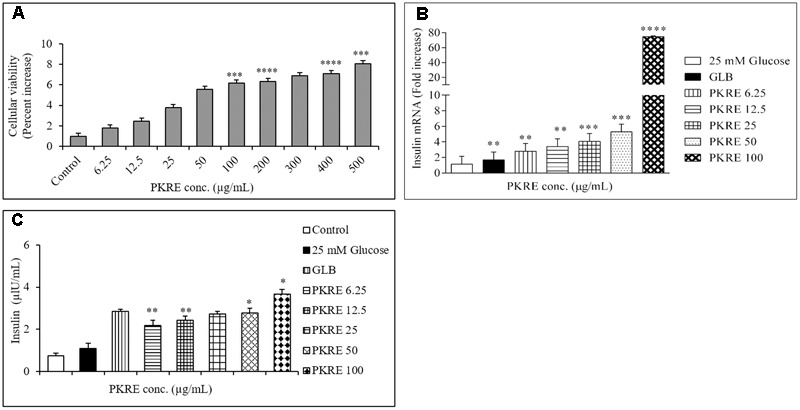
**(A)** Effect of PKRE on the viability of Rin5f cells. Rin5f cells were treated with indicated concentrations of PKRE (6.25, 12.5, 25, 50, 100, 200, 300, 400, and 500 μg/mL) for 48 h and viability was measured by SRB assay. **(B)** PKRE treatment also upregulated insulin transcript levels in Rin5f cells. Insulin fold change was normalized by *GAPDH* expression. Cells were treated with indicated concentration of PKRE (6.25, 12.5, 25, 50, and 100 μg/mL) or GLB for 90 min along with 25 mM KRB buffer. After incubation total RNA was isolated and qRT-PCR was performed by following manufacturer’s instruction. **(C)** PKRE enhanced the insulin secretion in Rin5f cells. Cells were treated with indicated concentrations of PKRE (6.25, 12.5, 25, 50 and 100 μg/mL) or GLB along with 25 mM glucose containing KRB buffer for 90 min. Secreted insulin in the supernatants were quantified by ELISA by following manufacturer’s instructions Experiments were done in triplicate on twice (*n* = 6). Data are expressed as mean ± SEM (*n* = 2; triplet of two separate experiments). ^∗∗^*p* < 0.01, ^∗∗∗^*p* < 0.001, and ^∗∗∗∗^*p* < 0.0001 indicate significant difference in groups compared to normal control group.

Cell viability measures the cytocompatibility as well as protective efficacy of test molecules. It enables to find the safe dose of a test component with minimal toxicity. Cellular viability on Rin5f cells was determined by SRB assay and we found that PKRE treatment significantly supports their proliferation in concentration dependent manner after 48 h exposure (**Figure 7A**). Further, non-lethal concentrations of PKRE significantly enhanced the insulin gene expression. Reduction in insulin levels results from destruction of *β*-cells in T1DM, which is directly linked with hyperglycemia and associated complications (Subash-Babu et al., 2015). PKRE and GLB significantly increases the transcript levels post 90 min treatment. It was found that PKRE significantly increases the transcript levels to 3.1 fold (*p* < 0.01) at 6.25 μg/mL as compared to high glucose control (1.16 fold) and GLB control (2.3 fold, *p* < 0.01). Further, insulin gene expression was upregulated to 3.4 (*p* < 0.01), 4 (*p* < 0.001), and 5.2 fold (*p* < 0.001) at 12.5, 25, and 50 μg/mL of PKRE treatment. However, highest transcript levels were recorded upto 74.7 fold (*p* < 0.0001) at 100 μg/mL (**Figure 7B**).

In order to further validate our above findings, cells were treated with 6.25, 12.5, 25, 50, and 100 μg/mL of PKRE for 90 min and the insulin secreted by the Rin5f cells were measured by ELISA. The insulin secretion was 2.1 μIU/mL (*p* < 0.01) when cells were treated at 6.25 μg/mL compared with high glucose control up to 1.1 μIU/mL. This was further found to be elevated in concentration dependent manner and could recorded as high as up to 3.68 μIU/mL (*p* < 0.05) at 100 μg/mL compared to GLB treated cells which recorded upto 2.8 μIU/mL (**Figure 7C**).

### PKRE Protects Cellular Viability and Enhances the Glucose Uptake by L6 and 3T3L1 Cells

**FIGURE 8:**
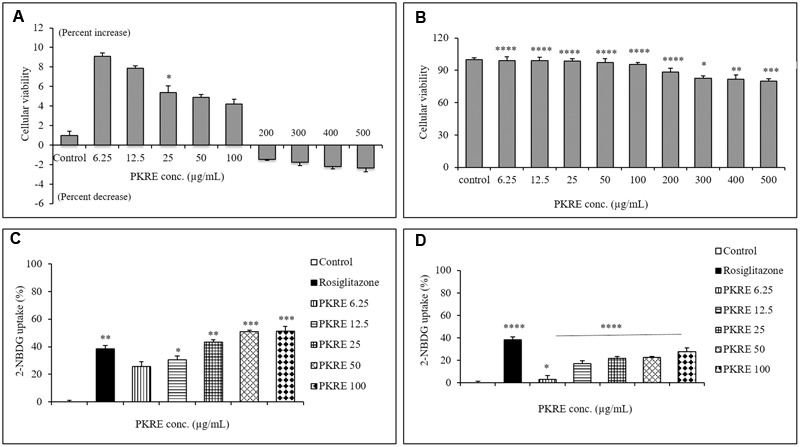
**(A)** Cytocompatibilty ability of different PKRE concentrations (6.25, 12.5, 25, 50, 100, 200, 300, 400, and 500 μg/mL) on the L6 cells viability was achieved by SRB assay after 48 h incubation. **(B)** PKRE treatment significantly also protected the viability of 3T3L1 cells after 48 h treatment, measured by SRB assay. **(C)** 2-NBDG measurement in L6 cells by flow cytometry. PKRE treatment significantly enhanced 2-NBDG uptake in differentiated myotubes. Mean fluorescence intensities were measured using Image Stream X MKII, Merck flow cytometer by acquiring single circular events. **(D)** Insulin stimulated 2-NBDG uptake was enhanced by PKRE treatment in differentiated 3T3L1 cells. Mean fluorescence intensities were measured using Image Stream X MKII, Merck flow cytometer by acquiring single circular events. Experiments were done in triplicate on twice (*n* = 6). Data are expressed as mean ± SEM (*n* = 6; triplet of two separate experiments). ^∗^*p* < 0.05, ^∗∗^*p* < 0.01, ^∗∗∗^*p* < 0.001, and ^∗∗∗∗^p < 0.0001 indicate significant difference in groups compared to normal untreated control group.

Cytocompatiblity of PKRE with L6 cells revealed that PKRE does not affect the cellular viability and in contrast supported the cellular proliferation upto 4.2% at 100 μg/mL concentration. However, we noticed decrease in cellular viability upto 2.3% with further increase in PKRE concentrations after 48 h exposure (**Figure 8A**). In 3T3L1 cells, we found that PKRE maintained the cellular integrity upto 95.5% (*p* < 0.001) at 100 μg/mL and further 79.9% (*p* < 0.001) viability was recorded at 500 μg/mL (**Figure 8B**). Afterward, sub-lethal concentration of PKRE were evaluated for their 2-NBDG uptake as a measure of glucose uptake potential in 3T3L1 and L6 cells. Insulin stimulated uptake in 3T3L1 and L6 cells significantly increases with PKRE treatment as compared to rosiglitazone. Rosiglitazone, a PPAR-γ agonist is an effective antidiabetic drug which modulates the secretory function of adipocytes and improves insulin sensitivity (Shyni et al., 2014). PKRE and rosiglitazone significantly enhances the glucose consumption in differentiated L6 cells. Mean fluorescence were measured in the cells treated with indicated concentration of PKRE (6.25, 12.5, 25, 50, and 100 μg/mL) and found elevated with increase in concentration. This could recorded highest upto 51.3%, (*p* < 0.001) at 100 μg/mL concentration after PKRE treatment (**Figure 8C**). In 3T3L1 cells, it was found that rosiglitazone increases the glucose uptake by 38.2% (*p* < 0.001) compared to control. The mean fluorescence levels in cells treated with 6.25, 12.5, 25, 50, and 100 μg/mL of PKRE were found increasing with concentration and recorded as high as upto 27.7% (*p* < 0.001) at 100 μg/mL (**Figure 8D**).

## Discussion

In the present study, phytomolecular profiling of PKRE revealed the presence of diverse metabolites belonging to different classes. We extensively performed the chemical characterization of the rhizome extract by 1D and 2D ^1^H-NMR metabolomic approaches and then quantified and validated the presence of major iridoid glycosides using HPTLC and UPLC DAD. This chemically characterized PKRE extract was further evaluated for OGTT, which revealed that PKRE effectively lowered the blood glucose level toward normalcy at both the treatment doses in the glucose loaded rats. These observations were taken further systematically to determine the efficacy of PKRE to ameliorate insulin insufficiency *in vivo* which was further validated at cellular and molecular level *in vitro*. Recently, Zahiruddin et al. (2016) reported the pharmacokinetics of iridoid enriched fraction of *P. kurroa* rhizome. Significant bioavailability of picroside I, II and apocynin, was detected in plasma and negligible amount was reported in urine reflecting its proper systemic consumption and metabolism. This provides the molecular basis for antihyperglycemic attributes of *P. kurroa in vivo*.

Pathophysiology due to T-cell mediated destruction of insulin producing *β*-cells leads to insulin shortage in the vital organs resulting in life threatening macro and micro vascular complications. Autoreactive lymphocytes, macrophages and cytokines such as IFN-γ and TNF-α also play prominent roles in pancreatic *β*-cells destruction (Padgett et al., 2013). In the background of above perspectives, the present study investigated the effect of PKRE on *β*-cell mass regeneration in STZ intoxicated rats and its effect on proliferation and insulin gene expression in Rin5f cells. Our data suggests that PKRE lowered the fasting blood glucose levels at both the treatment doses compared to STZ intoxicated rats. Blood glucose clearance was due to PKRE stimulatory effects on pancreas, which could lead to enhanced blood insulin levels and glut 4 translocation in the peripheral tissues. Increased insulin secretion is either due to the regeneration or by stimulating the existing pancreatic *β*-cells (Ong et al., 2011; Soltani et al., 2011). Expression analysis by IHC showed significant (*p* ≤ 0.05) increase in the insulin secretion by regenerating β-cells. Remarkably, PKRE also reduced *β*-cells apoptosis and reversed the insulin insufficiency, whereas STZ intoxicated group was marked by the presence of apoptotic bodies. Interestingly, proliferation of insulin producing Rin5f cells was observed in concentration dependent manner when cytocompatibilty of PKRE was evaluated. Transcript quantification and ELISA demonstrated that PKRE enhanced the insulin secretion by elevating the insulin transcript levels to levels as high as upto 74.7 fold.

It has been reported that Gcgr^-/-^ mice significantly reverses T1DM complications and also suggests that Gcgr antagonists as well as glucagon suppressors can be used as possible therapeutic agents to maintain blood glucose homeostasis (Lee et al., 2011). Our IHC results revealed the increased expression of Gcgr in STZ intoxicated group compared to normal untreated group and also throws light on the fact that PKRE effectively inhibits the hepatic and renal Gcgr expression, which further supported its anti-hyperglycemic property.

Altered levels of ALT, AST and ALP in the blood stream are the markers of hepatic dysfunction or integrity (Adefegha et al., 2014; Al-Malki and El Rabey, 2014). Imbalance in their levels is reported to be associated with the complications due to hyperglycemia (Arunachalam and Parimelazhagan, 2013) which was evident in STZ intoxicated group. However, PKRE administration effectively brought down these markers to their normal levels when compared with normal control animals. On the other hand, hyperglycemia significantly altered the levels of serum albumin, BUN and CK in STZ intoxicated group. Alterations in their levels are directly related to renal damage, which might be due to decreased protein synthesis and enhanced tissue proteolysis in diabetic rats (Nabi et al., 2013; Tzeng et al., 2013). Further we found that, PKRE increases the serum albumin while decreasing the BUN and CK levels toward normalcy in diabetic rats, indicating lower tissue proteolysis and normalized glomerular filtration. These results suggested that PKRE lowered the blood glucose and oxidative stress, which supported its protective effect against hyperglycemia mediated hepatic and renal damage.

Streptozotocin induced free radicals impaired the activity of antioxidant enzymes (SOD and CAT) with concurrently enhanced lipid peroxidation in STZ intoxicated rats compared to normal (Arunachalam and Parimelazhagan, 2013; Efiong et al., 2013). PKRE treatment activated SOD and CAT in both hepatic and renal tissues, respectively. Further, enhanced oxidative stress resulted in increased serum lipid peroxides due to oxidation of PUFA (Tangvarasittichai, 2015) as evident from STZ intoxication. PKRE also lowered the MDA levels to normal and restores the cellular oxidative status. Improvement in the oxidative stress may directly or indirectly protects *β*-cells from ROS mediated destruction, though the results are statistically not significant.

Skeletal muscles are considered as major site for the insulin stimulated glucose uptake as well as oxidation. Defective insulin sensitivity in skeletal muscle and adipose tissues leads to hyperglycemia (Ueda et al., 2012) Insulin stimulated glucose uptake is accompanied with glut 4 transporters, predominantly distributed in muscles and adipose tissues (Uldry and Thorens, 2004). In order to evaluate the effect of PKRE on insulin stimulated glucose uptake, we performed glucose uptake assays in L6 and 3T3L1 cells and compared their response with that of rosiglitazone. Our observations revealed that PKRE in varying concentrations significantly (*p* ≤ 0.05) enhanced the insulin stimulated glucose uptake in concentration dependent manner and supported the *in vivo* findings of enhanced insulin secretion from regenerated pancreatic *β*-cells.

Present study has provided insights for the presence of more than 31 major metabolites including iridoid glycosides in the PKRE. It exhibited *β*-cell regeneration potential as evidenced by IHC, which further complimented the enhanced insulin production. Additionally, results were validated *in vitro* on insulin producing Rin5f cells and it was found that PKRE proliferates *β*-cells at all treatment concentrations. It also results in elevated insulin gene expression and insulin secretion by Rin5f cells. Furthermore, PKRE was also found to enhance the insulin stimulated glucose uptake in 3T3L1 and L6 cells.

## Conclusion

In summary, present study demonstrated that metabolomically characterized hydro alcoholic extract of *P. kurroa* rhizome exerted strong *β*-cell regeneration potential along with enhanced insulin expression, antihyperglycemic effects and improves the hepatic and renal functions. Furthermore, PKRE possesses increased insulin stimulated glucose uptake potential but the underlying mechanism still needs to be explored in order to elucidate the insulin signaling in skeletal muscles and adipose tissues.

## Author Contributions

SK performed majority of the experiments. VP, SoS, SuS, KP, and DK assisted in performing different experiments. SK, VP, and DK analyzed and interpreted the data as well as drafted the manuscript. YP conceptualized, supervised the study and edited the manuscript. All authors contributed in the finalization of manuscript.

## Conflict of Interest Statement

The authors declare that the research was conducted in the absence of any commercial or financial relationships that could be construed as a potential conflict of interest.

## Abbreviations

ALP: alkaline phosphatase
ALT: alanine transaminase
AST: aspartate transaminase
ATCC: American type culture collection
BUN: blood urea nitrogen
CAT: catalase
CK: creatinine kinase
DMEM: Dulbecco’s modified eagles medium
FBS: fetal bovine serum
Gcgr: glucagon receptor expression
GLB: glibenclamide
MDA: malondialdehyde
NADH: nicotinamide adenine dinucleotide
NBT: nitroblue tetrazolium
OGTT: oral glucose tolerance test
PKRE: *Picrorhiza kurroa* rhizome extract
PMS: phenazine methosulphate
SOD: superoxide dismutase
SRB: sulforhodamine B
STZ: streptozotocin
T1DM: Type 1 diabetes mellitus
2NBDG: 2-deoxy-2-[(7-nitro-2,1,3-benzoxadiazol-4-yl)amino]-D-glucose
TBA: thiobarbituric acid
TCA: trichloroacetic acid
