# Supplementary material for: Picrorhiza kurroa Enhances β-Cell Mass Proliferation and Insulin Secretion in Streptozotocin Evoked β-Cell Damage in Rats
Source: Front Pharmacol. 2017 Aug 22;8:537. doi: 10.3389/fphar.2017.00537 (PMC5572391; doi:10.3389/fphar.2017.00537)
Supplement: Supplementary file 1 [file Data_Sheet_1.DOC]

Supplementary Material

***Picrorhiza kurroa* enhances *β*-cell mass proliferation and insulin secretion in streptozotocin evoked *β*-cell damage in rats**

Shiv Kumar^1,3#^, Vikram Patial^1#^, Sourabh Soni^1,3^, Supriya Sharma^1,3^, Kunal Pratap^1^ , Dinesh Kumar^2,3^*, Yogendra Padwad^1, 3^*

^1^Pharmacology and Toxicology Lab, Food and Nutraceutical Division, CSIR-Institute of Himalayan Bioresource Technology, Palampur, H.P., India

^2^Natural Product Chemistry and Process Development Division, CSIR-Institute of Himalayan Bioresource Technology, Palampur, H.P., India

^3^Academy of Scientific and Innovative Research, CSIR-Institute of Himalayan Bioresource Technology, Palampur, H.P., India

**# Authors contributed equally to this manuscript**

***Correspondence: Dr. Yogendra Padwad; Email:** [**yogendra@ihbt.res.in**](mailto:yogendra@ihbt.res.in) **and**

**Dr. Dinesh Kumar; Email:** [**dineshkumar@ihbt.res.in**](mailto:dineshkumar@ihbt.res.in)

**Running Title:** *β*-cell regeneration potential of PKRE

“CSIR-IHBT communication number for this manuscript is 4130”

**FIGURE S1** ^1^H-NMR spectra of PKRE, Mannitol, Picroside I and II **(a)**, Calibration curves of picroside I **(b)** and II **(c)**.


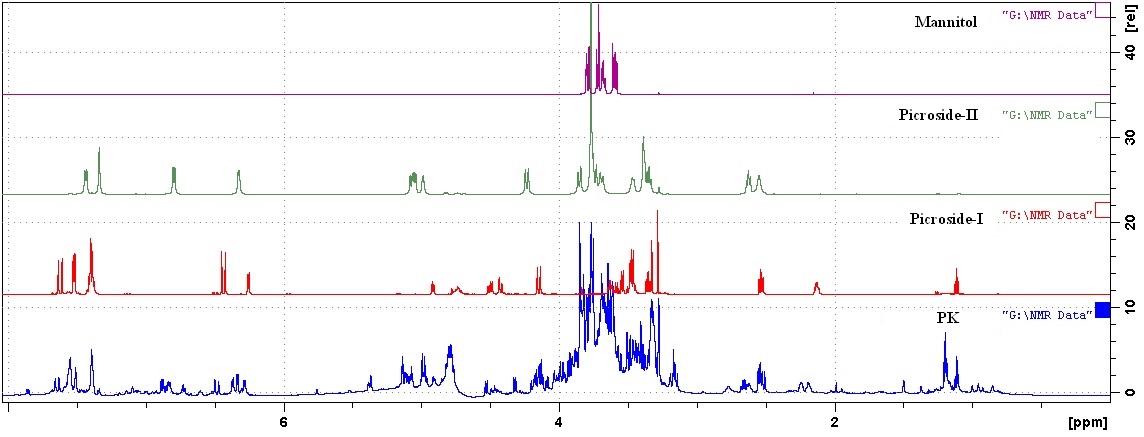


**(a)**


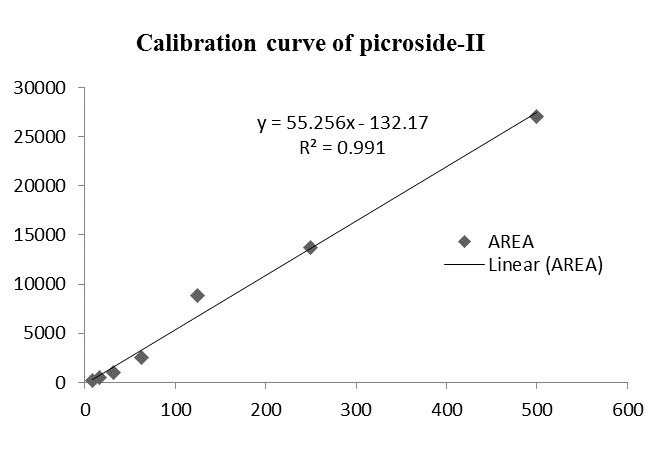


**(c)**


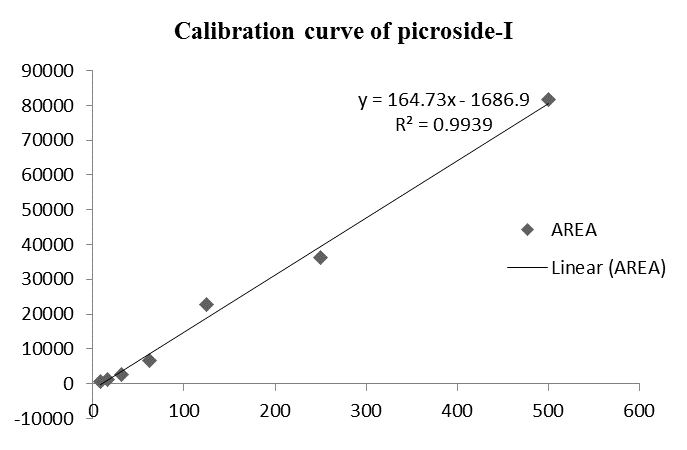


**(b)**

**TABLE S1** ^1^H-NMR based metabolites of PKRE

| **S. N.** | **Compound** | **Characteristic**  **Assignments/ correlations** | **^1^H (ppm)** | **^13^C (ppm)** |
| --- | --- | --- | --- | --- |
| **1** | ***β*-Glucose** | CH, 1 | 4.47 (d, 5.8 Hz) | 98.03 [1] |
| **2** | **α-Glucose** | CH, 1 | 5.11 (d, 3.7 Hz) | 94.16 [1] |
| **3** | **Fructose** | CH, 4 | 4.02 d (7.7 Hz) | 76.93 [[1] |
| **4** | **Mannitol** | Multiplets | *δ* 3.82 (dd, 2H) | *δ* 63.2, 69.7, 71.5 [2] |
| **5** | **Fatty acids** | CH3, terminal  (CH2–) n  (CH2–) n | 0.90 t (7.3 Hz)  1.29  1.33 | 14.8  35. [1]  34.8 |
| **6** | **Alanine** | *δ* 1.50 (d) | *δ* 3.76, 1.50 (d, *J* = 7.45) | [3] |
| **7** | **Valine** | *δ* 0.99 (d) | *δ* 0.99 (d, *J* = 6.24); *δ* 1.07 (d, *J* = 6.1). | [3] |
| **8** | **leucine** | *δ* 0.96 (d) | *δ* 0.96 (d, *J* = 7.5); 0.98 (d, *J* = 7.5) | [3] |
| **9** | **Isoleucine** | *δ* 0.94 (t) | *δ* 0.94 (t, *J*= 7.6) | [3] |
| **10** | **Glycine** | *δ* 3.55 (s) | *δ* 3.55 (s) | [3] |
| **11** | **Tyrosine** | *δ* 2.65 (t) | *δ* 2.15 (m) *δ* 2.65 (t, *J* = 8.14) | [3] |
| **12** | **Phenylalanine** | *δ* 7.43 | *δ* 7.38, *δ* 7.43 | [3] |
| **13** | **Androsin** | C4'-H | *δ* 3.78 | *β*-D-Glucopyranose 1-4'; *δ* 102.3, *δ* 74.2, *δ* 70.8, *δ* 77.9, *δ*  77.2, *δ* 61.6 (C1'-C6'), OCH_3_; *δ* 55.6  [4] |
| **14** | **Apocynin** | C4 –OH | *δ* 5.003 | OCH_3_; *δ* 55.6 [4] |
| **15** | **Picein** | *δ* 6.82 (dd) | *δ* 6.82 (dd) | C-2; *δ* 116.9, aliphatic carbon; *δ* 25.5 [5] |
| **16** | ***Trans*-hydroxy**  **cinnamic acids** | H-8 and H-7  (Olefinic protons) | Range from *δ* 6.41–6.53 and *δ* 7.52–7.70 | *δ* 167.1 (COOH), *δ* 146.6 (C-1), *δ* 140.4 (C-2), *δ* 104.8 (C-4), *δ* 128.1 (C-5) [6,7] |
| **17** | **Gallic acid** | *δ* 7.02, s. | *δ* 7.02, s. | [6,7] |
| **18** | **Syringic acid** | *δ* 3.88, s; *δ* 7.42, s. | *δ* 3.88, s; *δ* 7.42, s. | [6,7] |
| **19** | **Ferulic acid** | *δ* 3.87 (s) | *δ* 3.87 (OCH_3_) | [6,7] |
| **20** | ***Trans*-Cinnamic acid** | CH 7 and CH 5 | *δ* 6.46 (d), 7.44 (s) | [6,7] |
| **21** | **Vanillic acid** | *δ* 3.89 (s) | *δ* 7.56, 7.53, 6.82, (OCH_3_-3.89) | [6,7] |
| **22** | **Caffeic acid** | *δ* 6.46 (d), | *δ* 6.46 (d), 7.13, 7.19, 7.67 | *δ* C2'; 113.9 |
| **24** | **Shikimic acid** |  |  | *δ* 167.8 (COOH), *δ* 139.9 (C-2), *δ* 129.7 (C-1), *δ* 71.3 (C-4), *δ* 67.8 (C-5), *δ* 66.1 (C-3), *δ* 30.1 (C-6)  [8] |
| **25** | **Geranic acid** |  |  | C-1 to C-5 (C; *δ* 167.1, *δ* 115.2, *δ* 160.1, *δ* 41.8 and *δ* 25.5) **[9]** |
| **27** | **6-O-feruloyl catalpol Picroside III** | In HMBC: correlation of proton d, *δ* 3.65 with C *vs* O (d, *δ* 167.8), enabled us to assign the position of the feruloyl group | *δ* 3.87(3H s; ferulic acid), *δ* 3.48 and *δ* 7.69 dd; trans *α* & *β* protons of side chain attached with other molecule. *δ* 1.39 ppm indicating that hydroxyl group at C-6 is esterified with acid moiety | [10] |
| **28** | **Caffeoyl catalpol** |  | *δ* 7.09 (br. s), *δ* 6.82 (d); *δ* 7.12 (br. d); *δ* 7.54 (d); *δ* 6.27 (d); *δ* 7.12 (m); *δ* 7.51 (d); *δ* 6.41 (d) (*trans*-caffeoyl); *δ* 5.36 (1H, d,) and *δ* 4.85 (1H, d) anomeric protons signal of glucose; catalpol signals | [11] |
| **29** | **Picroside-I** | *trans*-Cinnamic acid, *α* & *β* unsaturation  In HMBC, the correlations, *δ* 4.34 d,) with C *vs* O (*δ* 167.1, d) enabled us to assign the position of cinnamoyl group | *δ* 4.50, 2H at C-6'  *δ* 6.47; d, (*J*= 15.9) and *δ* 7.84; d, (*J*= 8.65): catalpol signals | *δ* 147.5 and *δ* 120.5 [Standard comparison] |
| **30** | **6-O-vanilloyl catalpol and derivatives such as P-II** | In the HMBC, the correlations, H2-60 (d, *δ* 4.32) with C *vs* O (d, *δ* 167.1), enabled us to assign the position of the vanilloyl group. | *δ* 7.39 (d), *δ* 7.46 (dd, *J* = 1.8, 8.4 Hz), *δ* 6.82 (d, *J* = 8.46 Hz (3 aromatic protons); *δ* 8.43 (s) (1 phenolic hydroxyl proton); and *δ* 3.80 (s) OCH_3_ protons): vanilloyl ester part estrification at C-6. | [Standard comparison] |
| **31** | **Flavonoids** | Flavone  (H-8 with H-6 and H-5' with H-6' | H-8; *δ* 6.67, H-6; 6.40, H-5; *δ* 6.76 | Ring A at *δ* 124.2 (C-5), *δ* 128.1 (C-6), *δ* 133.8 (C-7), *δ* 120.5 (C-8). *δ* 151.4 (C-9), *δ* 120.6 (C-10), Ring B; C1'-6' at *δ* 131.1, 128.7, 129.7, 140.4, 128.9, 127.6, Ring C; C2-4 at *δ* 161.9, 112.6, 171.8  [12, 13, 14, 15] |

**References**

Porzel, A., Farag, M. A., Mulbradt, J., Wessjohann, L. A. (2014). Metabolite profiling and fingerprinting of *Hypericum Species*: A comparison of MS and NMR metabolomics. *Metabolomics.* 10, 574-588. DOI: 10.1007/s11306-013-0609-7

Gaidamauskas, E., Norkus, E., Vaiciūniene, J., Crans, D. C., Vuorinen, T., et al. (2005). Evidence of two-step deprotonation of D-mannitol in aqueous solution. *Carbohydr. Res*. 340, 1553-1556. doi: 10.1016/j.carres.2005.03.006

Mulas, G., Galaffu, M. G., Pretti, L., Nieddu, G., Mercenaro, L., et al. (2011). NMR analysis of seven selections of vermentino grape berry: Metabolites composition and development. J*. Agric. Food Chem.* 59, 793-802. doi: 10.1021/jf103285f

Huang, S., Liao, X., Nie, Q., Ding, L., Peng. S., (2004). Phenyl and phenylethyl glycosides from *Picrorhiza scrophulariiflora*. *Helv. Chim. Acta.* 87, 598-604.

Løkke, H. (1990). Picein and piceol concentrations in Norway spruce. *Ecotoxicol. Environ. Saf.* 19, 301-309.

Anastasiadi, M., Zira, A., Magiatis, P., Haroutounian, S. A., Skaltsounis, A. L., et al. (2009). ^1^H NMR-based metabonomics for the classification of Greek wines according to variety, region and vintage. Comparison with HPLC Data. *J. Agric. Food Chem.* 57, 11067-11074. DOI: 10.1021/jf902137e

Liu, M., Yang, S., Jin, L., Hu, D., Wu, Z., et al. (2012). Chemical constituents of the ethyl acetate extract of *Belamcanda chinensis* (L.) DC roots and their antitumor activities. *Molecules* 17, 6156-6169. doi: 10.3390/molecules17056156

Usuki, T., Yasuda, N., Yoshizawa-Fujita, M., Rikukawa, M. (2011). Extraction and isolation of shikimic acid from *Ginkgo biloba* leaves utilizing an ionic liquid that dissolves cellulose. *Chem. Commun. (Camb*). **47,** 10560-10562. doi: 10.1039/c1cc13306c

Crombie, L., King, R. W., Whiting, D. A. (1975). Carbon-13-magnetic resonance spectra. Synthetic presqualene esters, related cyclopropanes and isoprenoids. *J. Chem. Soc. Perkin. Trans.* 1, 913-915. doi: 10.1039/P19750000913

Young, H. S., Kim M. S., Park, H. J., Chung, H. Y., Choi, J. S. (1992). Phytochemical study on *Catalpa ovate*. *Arch. Pharm. Res.* 13, 322-327. doi: 10.1007/BF02974106

Compadre, C. M., Jáuregui, J. F., Nathan, P. J., Enríquez, R. G. (1982). Isolation of 6-O-(*p*-coumaroyl)-catalpol from *Tabebuia rosea*. *Planta Med.*  46, 42-44. doi: 10.1055/s-2007-970016

Owen, R. W., Haubner, R., Mier, W., Giacosa, A., Hull, W. E., et al. (2003). Isolation, structure elucidation and antioxidant potential of the major phenolic and flavonoid compounds in brined olive drupes. Food Chem. Toxicol. 41, 703-717.

Svehliková, V., Bennett, R. N., Mellon, F. A., Needs, P. W., Piacente, S., et al. (2004). Isolation, identification and stability of acylated derivatives of apigenin 7-*O*-glucoside from chamomile (*Chamomilla recutita* [L.] Rauschert). *Phytochem.* 65, 2323-2332. doi: 10.1016/j.phytochem.2004.07.011

Burns, D. C., Ellis, D. A., March, R. E*.* (2007). A predictive tool for assessing ^13^C NMR chemical shifts of flavonoids. *Magn. Reson. Chem.* 45, 835-845. doi: 10.1002/mrc.2054

Bochkov, D. V., Sysolyatin, S. V., Kalashnikov, A. I., Surmacheva, I. A. (2012). Shikimic acid: review of its analytical, isolation, and purification techniques from plant and microbial sources. *J. Chem. Biol.* 5, 5-17. doi: 10.1007/s12154-011-0064-8
